# A Novel Detection and Assessment Method for Operational Defects of Pipe Jacking Tunnel Based on 3D Longitudinal Deformation Curve: A Case Study

**DOI:** 10.3390/s22197648

**Published:** 2022-10-09

**Authors:** Wei Lin, Pan Li, Xiongyao Xie

**Affiliations:** 1 Department of Geotechnical Engineering, College of Civil Engineering, Tongji University, Shanghai 200092, China; 2 Key Laboratory of Geotechnical and Underground Engineering of the Ministry of Education, Tongji University, Shanghai 200092, China

**Keywords:** pipe jacking tunnel, defect, detection, assessment, longitudinal deformation, point cloud

## Abstract

Adjacent tunnel construction and environmental disturbances can lead to longitudinal deformation in pipe-jacking tunnels. The longitudinal deformation of the tunnel is closely related to the occurrence of joint dislocation, joint opening, and other defects. In view of the difficulty of obtaining 3D longitudinal deformation curves, a method is proposed to obtain 3D longitudinal deformation curves based on a large number of 3D point cloud data with high spatial resolution and large spatial dimensions. Combined with the mechanism of defects occurrence, a theoretical basis for tunnel defects assessment based on tunnel longitudinal deformation is proposed. Taking one pipe jacking tunnel as an example, the longitudinal settlement curve and the 3D longitudinal deformation curve are compared. The correlation between the 3D longitudinal deformation curve and defects such as mud leakage, cracks, and differential deformation is illustrated from the perspective of three indexes: deformation amount, bending deformation, and shearing deformation. The accuracy and reliability of the 3D longitudinal deformation curve in tunnel defects detection and assessment are verified.

## 1. Introduction

Different from structures with critical features such as buildings and bridges, tunnel structures are characterized by longitudinal linearity and fractal self-similarity. Proactive assessment of critical tunnel defects becomes a key issue in tunnel inspection-assessment-maintenance [1]. Since the longitudinal deformation and joint deformation, leakage, and other structural properties of tunnels are closely related to the diseases [2,3], the pre-determination of critical parts of diseases by longitudinal deformation curves becomes an important basis for tunnel detection-assessment-maintenance [4]. At present, the research on the relationship between longitudinal deformation and defect in tunnels focuses on 2D longitudinal deformation curves, especially settlement. Longitudinal settlement causes joint dislocation and opening, which will probably lead to stress concentration, lining damage, and stiffness loss, causing bolt failure and leakage [5,6]. In turn, the occurrence of leaks and other defects will promote consolidation settlement in the geotechnical body [7], triggering an increasing longitudinal differential settlement [8]. The longitudinal deformation of the tunnel is also an important controlling index in the seismic response analysis of the tunnel [9,10]. The longitudinal curve is closely related to joint status. Joint dislocation mainly occurs near the maximum curvature of the longitudinal settlement curve, and joint opening mainly occurs near the maximum value of the longitudinal settlement curve [8]. Tunnel convergence is concentrated near the inflection point of the longitudinal settlement curve [6]. However, the longitudinal settlement curve, which ignores the longitudinal horizontal deformation component, cannot completely characterize the longitudinal structural status of the tunnel. The 2D longitudinal settlement is actually the projection of the 3D tunnel deformation pattern in the vertical plane. Hence, the accuracy of the detection and assessment of key parts of tunnel defects cannot be guaranteed.

Longitudinal tunnel deformation includes not only longitudinal settlement, but also longitudinal horizontal displacement [11,12,13]. As well, horizontal displacement accounts for a significant proportion of the total longitudinal deformation [14]. In the 3D longitudinal deformation curve, tunnel joint dislocation and opening are closely related to the bending deformation and shearing deformation [15]. Bending deformation and shearing deformation have already been considered in existing analytical solutions for longitudinal deformation [16]. Due to the characteristics of long linearity, large cross-section, and buried underground, the current research on the relationship between 3D longitudinal deformation curves and defects in tunnels is limited to the analytical and numerical methods, and there is a lack of research on the relationship between 3D longitudinal actual measurement data and diseases. Chen et al. [17] stressed that all-around detection or monitoring is the development direction of the research for tunnel structural health. The establishment of a 3D longitudinal deformation curve based on the actual longitudinal measurements will improve the accuracy of tunnel structural performance assessment, which has important research significance and engineering practical value for tunnel structure detection, assessment, and maintenance [18].

For the detection of tunnel structural deformation, the mainly adopted equipment and methods include total station, static level, distributed fiber optic, and so on. Total station and static levels are simple to operate, have accurate results, and are easy to realize long-term automated monitoring [19,20]. Distributed fiber optic sensors have the advantage of full-range sensing [21,22]. However, the above methods can only obtain 2D longitudinal deformation curves due to the capability imitation. For direct detection of tunnel defects, there are some promising methods. Meniconi et al. [23] proposed a leak detection method based on Transient Test-Based Techniques (TTBTs), which shows great ability to directly sense the existence of water leakage. The main influencing features for TTBTs have been comprehensively studied [24]. Ultrasonic tomography (UST) is a nondestructive testing technology for the detection of impairments in tunnel lining [25,26]. For lining surface defect detection, an autonomous robot with computer vision system has been developed to inspect tunnel crack and efflorescence [27]. The deformation data, however, is not the main concern of these method. Comprehensive point cloud acquisition methods are also receiving attention, such as digital image correlation (DIC) and 3D laser scanning. DIC enables the measurement of strain fields and the identification of defect characteristics [28]. While the inspection methods based on DIC usually requires the installation of equipment inside tunnels. Three-dimensional laser scanning technology, which can quickly obtain a large amount of point cloud data in the three-dimensional space of tunnel structure, is used to analyze tunnel structure, such as convergence [29], limiting boundary [30], water leakage [31], and falling blocks [32]. An error model of laser scanning have been used to analyze the spatial distribution of the errors in a point cloud of a circular tunnel section, which shows that the errors induced by the angle of incidence will be eliminated when it comes to surface fitting [33]. The optimal positions of the scanner throughout the tunnel can be calculated based on the point density [34]. Xie et al. [35,36] investigated the measurement method of 3D laser scanning technology in full-section deformation of tunnel structures. The ellipticity of tunnel sections measured based on 3D laser scanning point cloud can be analyzed as an index for operational tunnel health status [37]. The 3D laser scanning technology has shown the advantages in the detection of local tunnel deformation, but the relationship between large amount of point cloud data and tunnel structural performance needs further to be researched. At present, 3D laser scanning technology has not been found to be applied in the analysis of 3D longitudinal deformation curves of tunnels and tunnel joint status.

To address the challenges of accurate detection and assessment of tunnel structural defects, this study proposes a novel method for measuring the longitudinal 3D deformation curve (3D-LDC) of tunnels based on 3D laser scanning point cloud data. The main contents of this study include: (1) the calculation method of 3D-LDC and tunnel structural defects based on 3D laser scanning technology; (2) the comparison analysis of the measured 2D longitudinal deformation curve (2D-LDC) and 3D-LDC; (3) the detection and assessment method of key defects and corresponding index based on 3D-LDC.

## 2. Site Information

Pipe-jacking method is one kind of tunnel construction method in soil ground, consists of jacking segmental pipes by means of hydraulic jacks to complete underground network. Due to the segmental structure, there will be joints connecting lining rings. Joints are design to bear the loads and should have the water-proof capacity. However, in the tunnel service phase, which is designed to be 100 or 120 years usually, the soil and water conditions will suffer from the disturbance of adjacent construction or inner evolution. Hence the performance of tunnel structure will be affected and then kinds of tunnel defects will occur.

**Figure 1:**
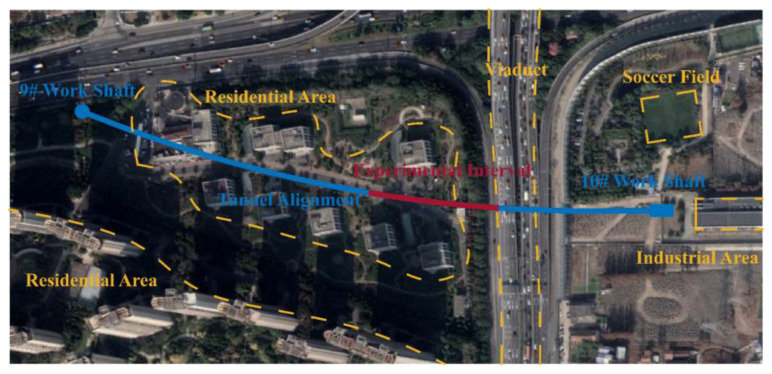
Plan view of a power transmission tunnel interval between 9#~10# work shaft.

A power transmission tunnel in Shanghai constructed by concrete pipe jacking has a total length of 2780 m. The inner diameter of pipe jacking tunnel is 3000 mm, with the lining thickness of 270 mm and ring length of 2.5 m. Deformation joints were set for every 15 rings in the tunnel. Figure 1 shows the plan view of the tunnel interval between 9# and 10# work shaft, which is 530 m long. The burial depth of the tunnel between 9# and 10# working shaft are 5.68 m and 6.23 m respectively, with a slope of 0.1%.

**Figure 2:**
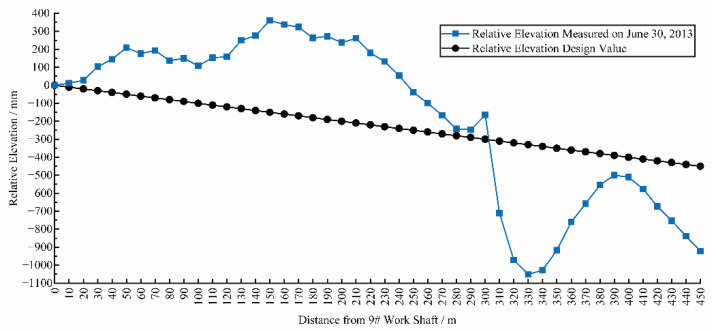
Longitudinal relative elevation curve of the tunnel on 30 June 2013.

In May 2013, a large disengagement between pavement and the tunnel lining, occurring between 290 m and 310 m from work shaft 9# (i.e., Ring 116 to Ring 124), was revealed during a field tunnel inspection. The maximum amount of disengagement, up to 80 mm, appeared at 295 m from the 9# work shaft (i.e., Ring 118). As well, cracks were found in the concrete pavement. The joint openings between 290 m and 330 m from 9# work shaft (i.e., Ring 116 to Ring 132). The multilayer composite plate liner had fallen off. At the same time, the longitudinal settlement curve showed that significant deformation had occurred, as depicted in Figure 2.

In March 2021, a mud leak was found in the tunnel during inspection. Emergency repairing was carried out using polyurethane, and the leakage had been sealed. Although the sealing was finally completed, the exact location of the mud leakage could not be accurately judged during the repair process due to the obscuration of the mud, which seriously affected the repair efficiency and effect.

## 3. Methodology

### 3.1. Assessment Theory of Defect Location Based on 3D-LDC

This subsection descripts the theory of tunnel defect assessment method based on 3D-LDC. The main tunnel defects include deformation-induced cracks and joint failures, as well as consequent water leakage and falling block. The magnitude and direction of displacement, the curvature and normal direction of tunnel axis, and the amount of joint shearing deformation, can be extracted from 3D-LDC, as the indexes for tunnel defect assessment.

Geotechnical-structural interaction is the most important factor affecting the structural performance of tunnels. On the one hand, geotechnical loading and unloading will cause displacement of the geotechnical body and structure. In turn, the displacement of the tunnel will change the geotechnical-structural interaction. In addition, the location of the largest tunnel deformation is often where the tunnel is closest to the external loads.

Cracks, as one of the main tunnel defects, may occur on the tunnel lining structure or on the subsidiary structures inside the tunnel. Cracks on the lining are mainly related to the lining bending deformation. Because of the positive correlation between lining strain *ε_t_* and curvature *κ*, the occurrence of cracks can be judged by the magnitude of curvature. The relationship between lining strain and curvature can be expressed as an equation:

(1) $$\varepsilon_{t}=y_{t}\kappa ,$$

where *y_t_* is the distance from the edge of the tensile zone to the neutral axis in the section. Cracks occur when the lining strain exceeds the maximum tensile strain that the lining material can withstand. Similarly, the relationship between lining strain and joint opening angle *θ* can be expressed as an equation:

(2) $$\varepsilon_{t}=\frac{y_{t}}{2\theta }.$$

**Figure 3:**
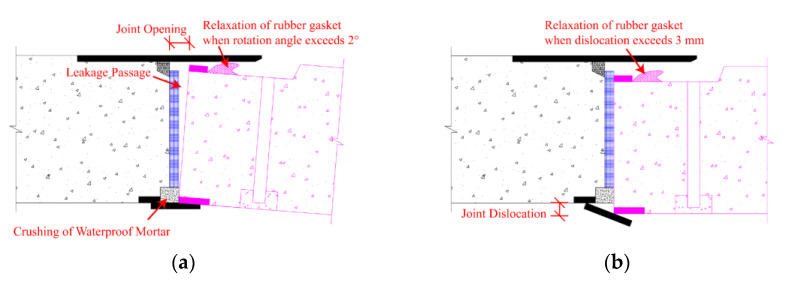
Schematic diagram of F-shaped joint water-proof failure due to longitudinal deformation: (**a**) Joint water-proof failure due to bending deformation; (**b**) Joint water-proof failure due to shearing deformation.

The 3D longitudinal deformation of the tunnel is reflected in the joints as opening and dislocation. From mathematical perspective, the integration of the joint opening and dislocation equals the longitudinal deformation. In turn, the differential of the longitudinal deformation at the joint is the opening and dislocation. Failure of the joint may occur when joint opening and dislocation exceed the design capability, resulting in water leakage. Taking the F-shaped joint of the pipe jacking tunnel as an example, the joint failure modes are shown in Figure 3. Bending deformation and shearing deformation of the tunnel in the longitudinal direction will lead to the relaxation of rubber gasket or the crushing of waterproof mortar, which in turn can lead to joint water-proof failure. Therefore, the 3D LDC of the tunnel can be used to judge the water-proof performance and the occurrence of defect.

### 3.2. Data Acquisition and Pre-Processing Methods

The Terrestrial Laser Scanner (TLS) can be used to obtain high precision point cloud data. The core technology of the TLS is the LiDAR technique, which is used to obtain the distance of each object point from the lens. The acronym LiDAR stands for light detection and ranging. The laser system produces and emits a beam (or a pulse series) of highly collimated, directional, coherent, and in-phase electromagnetic radiation. When the light reflected by the surface of an object is received, the system can calculate the range by the flight time and acquire the reflectivity of the surface. Regardless of the increasing of the laser’s spot dimension, the scanner records the center of the spot as a point; therefore, the point density is lower than 1 mm even in the maximum distance.

The TLS used in this paper is Leica ScanStation P40, of which the technical parameters are shown in Table 1. It should be noted that the method proposed in this paper does not rely on TLS only. Point cloud data collected by other devices that meet the accuracy requirements can also be used for the proposed method, such as mobile 3D laser scanner, LiDAR and depth camera.

The data acquisition steps are listed as follows: (a) Determine the station distance and the number of stations n according to the tunnel size and inspection area; (b) Layout the target between the *i*-th and (*i* + 1)-th stations (*i* = 1, 2, 3, …, *n* − 1); (c) Carry out the 3D laser scanning of the *i*-th station; (d) Carry out the 3D laser scanning of the *n*-th station.

The data pre-processing steps are listed as follows: (a) Extract the target coordinates; (b) Realize the alignment of cloud data from adjacent stations according to the target coordinates; (c) Perform coordinate conversion according to the relationship between the point cloud coordinate system and the world coordinate system; (d) Remove the noise point clouds generated by other objects in the tunnel; (e) Identify the point clouds of the tunnel lining and joints.

There are some existing point cloud processing solutions and software, such as Leica Cyclone and CloudCompare, of which the above mentioned pre-processing methods are built-in features. Hence the proposed pre-processing procedure can be carried out feasibly. Related pre-processing methods include target locating, alignment, segmentation of point cloud. The adopted laser scanner offers target coordination with accuracy of 2 mm @ 50 m. Taking CloudCompare, an open source project, for example, the alignment, noise removal, segmentation can be operated in the graphic interactive way, which is easy to conduct. Noise removal will help improve the robust of the following calculation procedure, due to the elimination of disturbance from noise points.

### 3.3. 3D Longitudinal Deformation Calculation Method Based on Point Cloud

The 3D-LDC of the tunnel can be calculated from the continuously distributed point cloud data of the tunnel. A local coordinate system is established at the tunnel center point, then the longitudinal 3D deformation curve can be expressed as:

(3) $$u=\int_{w_{1}}^{w_{2}}\frac{du}{dw}dw,$$

(4) $$v=\int_{w_{1}}^{w_{2}}\frac{dv}{dw}dw,$$

where *u* is the displacement of the tunnel center point in the horizontal direction, *v* is the displacement of the tunnel center point in the vertical direction, and w is the distance from the tunnel center point along the longitudinal direction to the center of the starting ring within the inspection interval.

Intercepting the measurement unit along the longitudinal direction of the tunnel, the displacement can be approximated as linear to the mileage in a sufficiently small range, and then Equations (3) and (4) can be expressed respectively as:

(5) $$u=\int_{w_{1}}^{w_{2}}f\left(w\right)dw=\int_{w_{1}}^{w_{2}}\left(a_{u}w+b_{u}\right)dw,$$

(6) $$v=\int_{w_{1}}^{w_{2}}g\left(w\right)dw=\int_{w_{1}}^{w_{2}}\left(a_{v}w+b_{v}\right)dw,$$

where *f*(*w*) and *g*(*w*) are linear function, and *a_u_*, *b_u_*, *a_v_* and *b_v_* are undetermined coefficients. For assembled tunnels such as pipe jacking and shield tunnels, each ring can be divided as one measurement unit. For mountain tunnels, a relatively smaller length can be intercepted as a measurement unit depending on the deformation.

**Figure 4:**
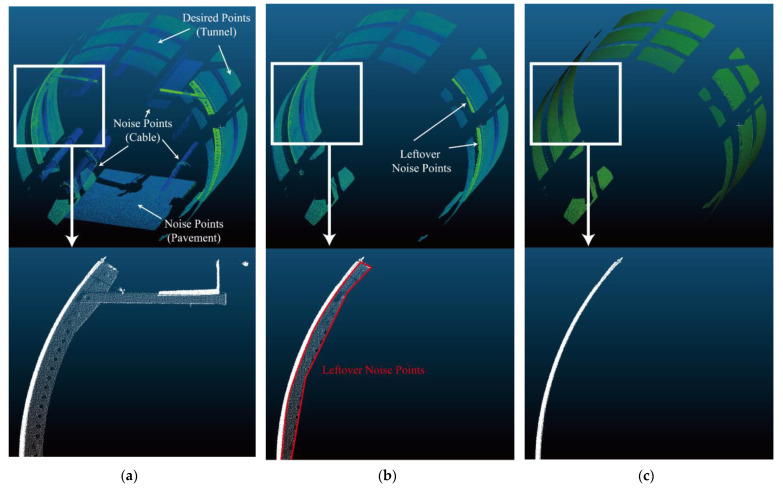
Noise removal result of segmented point cloud: (**a**) Raw segmented point cloud of one tunnel ring; (**b**) Noise points removed by manual operation; (**c**) Leftover noise points removed by RANSAC.

The random sample consensus (RANSAC) algorithm is used for cylindrical fitting of point set *P_j_* within the measurement unit, which can reflect the 3D longitudinal attitude of tunnel. There may be some leftover noise points which are difficult to determine in the pre-processing, due to the limitation of manual operation. Different from direct cylindrical fitting based on least squares only, RANSAC enables the further filtration of leftover noise points. As well, the parameters of fitted cylinder, including coordinates of endpoints and diameter, will also be determined by using RANSAC [36,38]. The steps are listed as follows: (a) Select the smallest data set for which the model can be estimated; (b) Use the data set to calculate the model; (c) Bring all the data into the model and calculate the number of interior points (accumulate the data that fit the model in the current iteration within a certain error range); (d) Compare the number of interior points of the current model with the best model obtained before, and record the model parameters and the number of interior points; (e) Repeat the above four steps until the end of the iteration or the current model is good enough (the number of interior points is greater than a certain number). An actual example can be found in Figure 4. Figure 4 depicts the removal results of noise points by applying RANSAC in proposed workflow. Manual removal operation is feasible for obvious noise points. When the noise points are close to desired points and there is no distinct boundary between them, the manual removal operation will be hard to carry out. By applying RANSAC, the leftover noise points will be removed in the iterative fitting loops. In the case of provided example, raw point cloud, manual edited point cloud and the final point cloud contains 436,133, 284,541 and 264,404 points respectively. In raw point cloud, about 39.4% points are useless and of disturbance for deformation detection.

LDC calculation can be performed based on the results of the above longitudinal deformation calculation. The curvature of the quadratic curve determined by three adjacent axial endpoints is used as the estimated curvature, carried out in the horizontal and vertical planes respectively. Three points *Q*_1_(*u*_1_ *v*_1_ *w*_1_)^T^, *Q*_2_(*u*_2_ *v*_2_ *w*_2_)^T^ and *Q*_3_(*u*_3_ *v*_3_ *w*_3_)^T^ are assumed to be endpoints obtained from adjacent measurement units. The estimated curvature in the horizontal plane *κ_h_* at *Q*_2_ can be calculated as:

(7) $$\kappa_{h}=\frac{2\left(a_{3}b_{2}-a_{2}b_{3}\right)}{{\left(a_{2}^{2}+b_{2}^{2}\right)}^{\frac{3}{2}}},$$

(8) $$\{\begin{array}{c} A={\left(\begin{array}{ccc} a_{1} & a_{2} & a_{3} \end{array}\right)}^{T}=M^{-1}U\\ B={\left(\begin{array}{ccc} b_{1} & b_{2} & b_{3} \end{array}\right)}^{T}=M^{-1}W \end{array},$$

(9) $$M=\left(\begin{array}{ccc} 1 & -t_{12} & t_{12}^{2}\\ 1 & 0 & 0\\ 1 & t_{23} & t_{23}^{2} \end{array}\right),$$

(10) $$\{\begin{array}{c} t_{12}=\sqrt{{\left(u_{2}-u_{1}\right)}^{2}+{\left(w_{2}-w_{1}\right)}^{2}}\\ t_{23}=\sqrt{{\left(u_{3}-u_{2}\right)}^{2}+{\left(w_{3}-w_{2}\right)}^{2}} \end{array},$$

(11) $$\{\begin{array}{c} U={\left(\begin{array}{ccc} u_{1} & u_{2} & u_{3} \end{array}\right)}^{T}\\ W={\left(\begin{array}{ccc} w_{1} & w_{2} & w_{3} \end{array}\right)}^{T} \end{array},$$

where *a*_1_, *a*_2_, *a*_3_, *b*_1_, *b*_2_ and *b*_3_ are undetermined coefficients, and *t*_12_ and *t*_12_ are the distance of *Q*_1_ to *Q*_2_ and *Q*_2_ to *Q*_3_ respectively. Similarly, the curvature *κ_v_* in the vertical plane can be calculated.

For the assembled tunnels, the longitudinal rotation of tunnel lining is mainly concentrated at the joints. Therefore, there is approximated correspondence between the joint opening angle and the curvature. In practical applications, the bending deformation can also be characterized by measuring the relative rotation angle between the measurement units. For a certain length of measurement unit, the relationship between the turning angle *θ* and the curvature *κ* is shown as:

(12) $$\theta =\frac{1}{2\kappa }.$$

The amount of joint dislocation can also be calculated on the same theory. *Q_j_*_2_(*u_j_*_2_ *v_j_*_2_ *w_j_*_2_)^T^ and *Q*_(*j*+1)1_(*u*_(*j*+1)1_ *v*_(*j*+1)1_ *w*_(*j*+1)1_)^T^ are assumed to be the endpoint of *j*-th ring joint (between the *j*-th ring and the *j* + 1 − *t* ring). The horizontal dislocation Δ*u* and vertical dislocation Δ*v* of the *j*-th joint can be calculated as:

(13) $$\{\begin{array}{c} \Delta u=u_{\left(j+1\right)1}-u_{j2}\\ \Delta v=v_{\left(j+1\right)1}-v_{j2} \end{array}.$$

### 3.4. Workflow of Proposed Method

**Figure 5:**
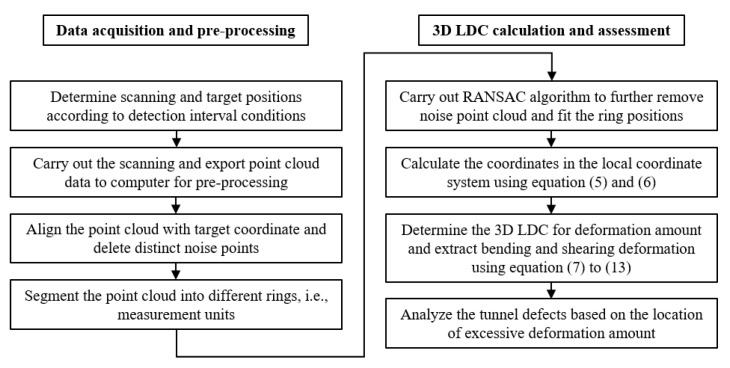
Workflow of proposed method.

The workflow of proposed method is shown in Figure 5. The whole procedure can be divided into two main parts. Data acquisition will be carried out in the field and the others can be conducted in the office. Pre-processing is carried out after the point cloud is collected to ensure the feasibility and robust of following 3D LDC calculation. The pre-processing steps include alignment, segmentation of point cloud and the removal of noise points. Then the RANSAC will be applied in the point cloud for the purposes of both noise removal and cylinder fitting. The 3D LDC can be calculated based on the fitted results. The more detailed deformation indexes then can be extracted from 3D LDC for further assessment of tunnel defects.

## 4. Field Experiment

### 4.1. Overview of Field Experiment

**Figure 6:**
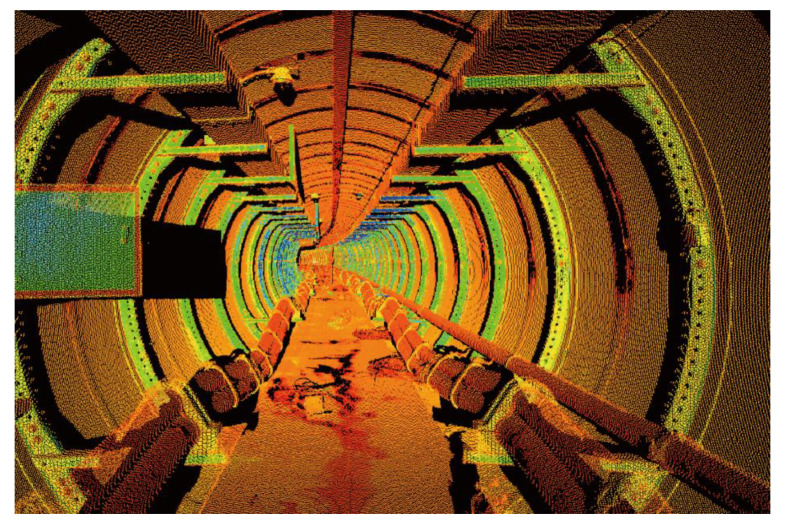
3D point cloud obtained from a field experiment.

**Figure 7:**
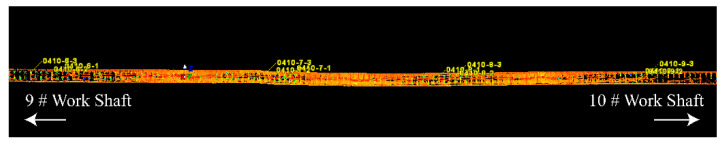
3D point cloud alignment results of the provided case.

In this paper, a field experiment has been conducted for the interval with severe tunnel deformation and defects, i.e., from Ring 105 to Ring 153. TLS was used to collect 3D point clouds from Ring 105 to Ring 153, with 5 stations. Figure 6 shows the 3D point clouds obtained by TLS. All the objects in the tunnel have been scanned in high density. In Figure 6, the RGB value stands for the intensity of each point, which can be used as characteristic for distinct different object. The coordinates of the target are recorded between two adjacent stations, which are used to realize the alignment for point cloud of all stations. Figure 7 shows the final obtained 3D point cloud alignment results in the provided case, which contains 5 scanning stations and 66,019,160 points. The scanning resolution is 0.057°. Among the aligned point cloud, point cloud of 48 tunnel rings is segmented for further analysis. For each segmented point cloud of tunnel rings, as shown in Figure 4a, the workflow of pre-processing and 3D LDC calculation is carried out.

The pipe jacking tunnel is one type of assembled tunnel with a large difference in stiffness between the joint and the lining, which allows each ring to be assumed as a measurement unit. Programs for data processing are coded in Python. A total station was used for elevation measurement to verify and compare the 3D laser scanning measurement results. The coordinates of reference points were recorded using total station and TLS. The data measured by both methods were converted under the local coordinate system. The local coordinate system was defined as a right-handed spiral coordinate system, with v-axis representing vertically upwards, w-axis representing the tunnel forwarding direction and u-axis representing the right side of the tunnel forwarding direction.

### 4.2. Results of Field Experiment

**Figure 8:**
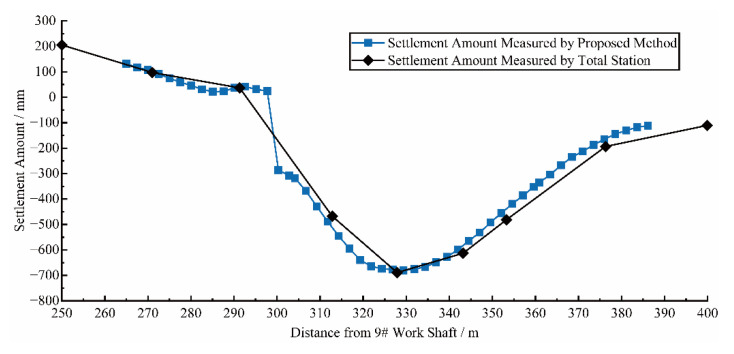
Comparison of experimental results of vertical LDC.

Vertical LDP, representing settlement, obtained by using the proposed methods and the total station are compared, as shown in Figure 8. The trends and values of both are very close to each other. It can be seen that the longitudinal deformation measurement method proposed in this paper achieves high accuracy. As well, due to the improvement of data acquisition efficiency, the proposed method can significantly improve the spatial resolution of the longitudinal deformation measurement results. Among them, there is a large local differential settlement around 300 m from the 9# work shaft. Due to the large spacing of level measurement, this information was not captured by the total station. In contrast, the 3D point cloud-based measurement method is able to obtain the deformation data of each ring and provide a comprehensive result of the longitudinal deformation, showing the capability of the proposed method to capture local deformation characteristics.

**Figure 9:**
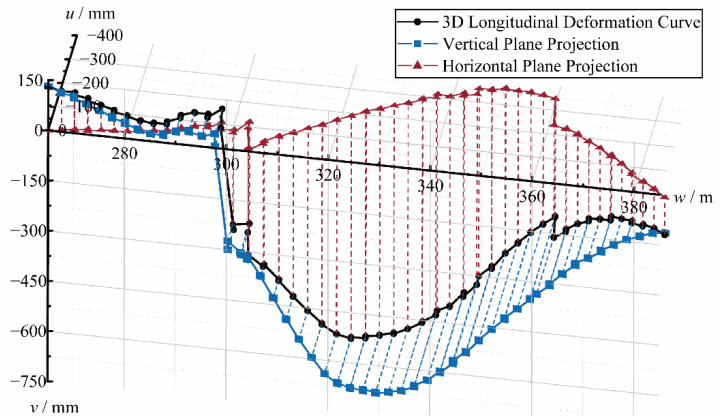
Experimental results of 3D-LDC.

Compared with the traditional tunnel longitudinal deformation measurement method, the proposed method not only has a higher spatial resolution, but also can obtain more dimensional data, i.e., 3D-LDC. 3D-LDC shows that the tunnel is deformed in different directions in space, as shown in Figure 9. The 3D-LDC and its projections in the horizontal and vertical planes are shown in Figure 9. The orange line indicates the horizontal LDC and the cyan line indicates the vertical LDC. It can be seen that the longitudinal deformation is dominated by the vertical deformation, which reaches a maximum of 679 mm and occurs at Ring 129. While the deformation in the horizontal plane is also not negligible, reaching a maximum of 369 mm, which occurs at Ring 138. The maximum value of 3D LDC appears in the middle of the above two, reaching a maximum of 727 mm at rings 132 and 133. From the perspective of deformation distribution, both the total and the fractional longitudinal deformation of the tunnel show characteristics of large middle and small sides in the experimental interval. Table 2 lists the maximum values of each index of 3D LDC and corresponding locations.

## 5. Tunnel Defect Assessment

Tunnels are buried underground, resulting in difficulty in observing and determining the location of the defect. This section illustrates the accuracy and advantage of 3D-LDC indexes in tunnel defect assessment through the comparison of 3D LDC and vertical LDC.

### 5.1. Defect Assessment Based on Deformation Amount

**Figure 10:**
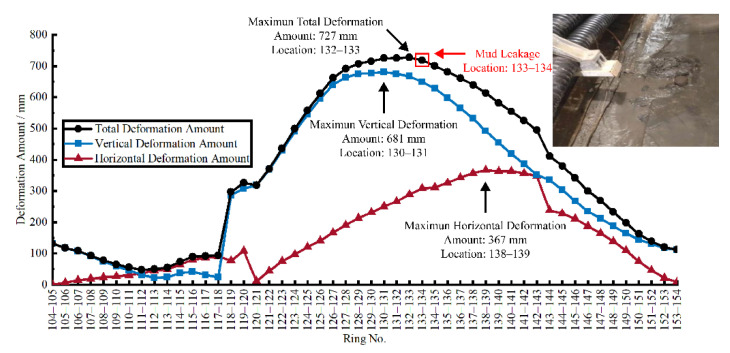
Correlation between the amount of deformation and tunnel defect.

Figure 10 shows the amount of longitudinal horizontal deformation, settlement, and total deformation of the tunnel. From Table 2 and Figure 10, the 3D-LDC shows that the maximum deformation of 727 mm occurs at joints 132–133. The vertical LDC shows that the maximum vertical deformation of 681mm occurs at joints 130–131. In addition, the maximum horizontal deformation of 367 mm occurred at joints 138–139. The field defect investigation showed that the mud leakage occurred at joints 133–134. The distance between the location of mud leakage and the location of maximum total deformation was only 1 ring (2.5 m). The distance between the location of mud leakage and the maximum vertical deformation location and the maximum horizontal deformation location is three rings (7.5 m) and five rings (12.5 m), respectively. It indicates that longitudinal vertical deformation and horizontal deformation are both related to the occurrence of mud leakage. There is a significant correlation between the total deformation and the occurrence of mud leakage. Since the vertical LDC only represents deformation in the vertical plane, the accuracy of judging the mud leakage location by it only is low.

### 5.2. Defect Assessment Based on Bending Deformation

**Figure 11:**
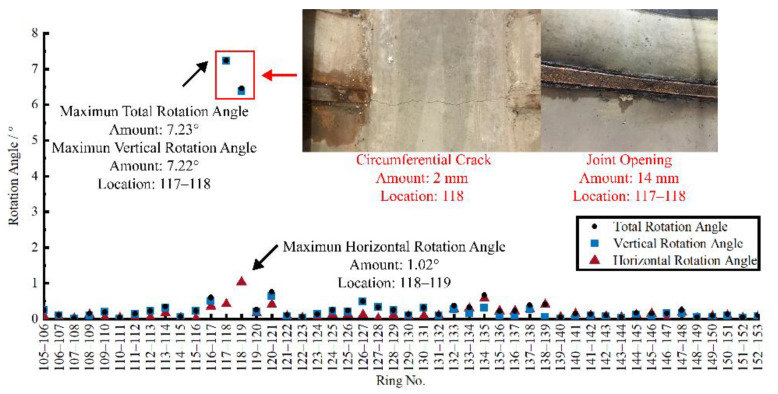
Correlation between the bending deformation and tunnel defect.

The rotation angle between the measurement units can reflect the longitudinal bending deformation of the tunnel. According to the curvature calculation method mentioned in Section 3.3, the angles between the measurement units in the experimental area were calculated based on the 3D-LDC in Section 4.2, and the results are shown in Figure 11. In terms of the total and vertical rotation angle, the largest values were found at joints 117–118, and slightly smaller at joints 118–119. The field defect investigation found that a 2mm circumferential crack appeared in the pavement bedding at ring 118. It can be speculated that the larger bending deformation at this location triggered larger additional stress in the tunnel subsidiary structure, leading to the appearance of the circumferential crack. At the same time, the joint openings at the top of the tunnel at joints 117–118 and 118–119 reached 14 mm and 11 mm, respectively. The joint openings further led to the occurrence of leakage and corrosion of the steel at the joints. According to the safety limitation of bending deformation given by Ye et al. [39], which is 1.022° for a pipe jacking tunnel with an outer diameter of 3540 mm and ring length of 2.5 m, the bending deformation of joint 117–118 has already exceeded the safety limitation. Exceeding safety limitations resulted in the occurrence of the tunnel defects mentioned above. As well, the bending deformation of joints 118–119 is around the limitation, which results in the obvious joint opening. In addition, the largest horizontal rotation angle appeared at joints 118–119, showing a clear correlation with the occurrence of the defect. It is worth mentioning that, according to the field disease investigation, no cracks or joint openings were found in other locations within this experimental interval, which complied with the calculated results of smaller turning angles in other locations. In other joints of the excremental interval, no exceeding of safety limitation is found. From the above analysis, it is clear that the 3D-LDC-derived index rotation angle can be used to judge the bending deformation of the tunnel and the location of the tunnel defects caused by bending.

### 5.3. Defect Assessment Based on Shearing Deformation

**Figure 12:**
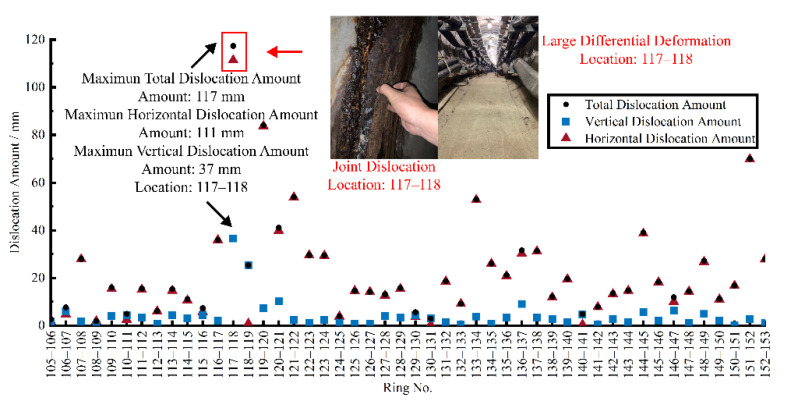
Correlation between the shearing deformation and tunnel defect.

The joint dislocation is a reflection of the longitudinal tunnel shearing deformation. According to the calculation method of dislocation proposed in Section 3.3, the joint dislocation between the measurement units in the experimental area was calculated based on the 3D LDC in Section 4.2, and the results are shown in Figure 12. Similar to the rotation angle, the joint dislocation also occurred near joints 117–118. The field defect investigation revealed that a large joint dislocation appeared on the side of the tunnel at joints 117–118, which is consistent with the calculated results. In addition, the bending deformation and shearing deformation together caused the undulation of the pavement in the tunnel, affecting the tunnel’s performance. Different from bending deformation, horizontal dislocation has a larger contribution to total dislocation. It can be found that for the experimental interval, the vertical deformation is mainly triggered by bending deformation and the horizontal deformation is mainly triggered by shearing deformation.

## 6. Discussion and Conclusions

### 6.1. Discussion

(1) This paper verifies the feasibility and accuracy of the proposed method in a pipe jacking tunnel, which has a broad application prospect. However, the number of defects used to validate the proposed method is limited due to the uncommon occurrence of severe defects in tunnel projects in practice. In addition, the 3D-LDC measurement method for tunnels proposed in this paper is theoretically applicable to various structural forms of tunnels, such as shield tunnels and rock tunnels. It can be subsequently verified by testing more engineering practices.
(2) A tunnel is a structure buried in rock and soil, and there are hidden surfaces that cannot be measured. As a result, some deformation indexes, such as joint dislocation, are difficult to be measured directly. Therefore, some indexes in this paper have not been verified by other measurement methods.
(3) The advantages of 3D laser scanning incldeu fast non-contact data acquisition procedure and a huge amount of acquired data. One station of scanning, which takes less than 5 minutes, will cover tens of meters, depending on tunnel diameter. The non-contact scanning procedure allows the power transmission tunnel to function normally during the detection and assessment. The point cloud data is distributed in three-dimensional space, which is hard to be realized by other methods, such as distributed fiber optic sensors and static hydraulic levels. The whole scanning process is automatic, which is not possible for a traditional method using a total station. The process of 3D laser scanning point cloud data processing, however, is still time-consuming and requires improvement in automatic processing. As well, the measurement method based on a 3D point cloud is to be improved for the measurement of local deformation at the joint.
(4) For 3D-LDC, more detailed criteria for disease determination can be proposed through theoretical analysis and numerical simulation, and. The 3D-LDC can also be combined with the information model to visualize the tunnel performance in a more intuitive way.

### 6.2. Conclusions

(1) The inspection method proposed in this paper can improve the acquisition efficiency and spatial resolution of tunnel deformation measurement results, and obtain high accuracy of 3D-LDC. 3D-LDC provided in this paper has higher spatial resolution and larger spatial dimension, which includes the spatial deformation data of any part of the tunnel and can obtain various deformation indexes in 3D space.
(2) This paper illustrates the mechanism of tunnel disease and longitudinal deformation, and proposes the theoretical basis of 3D-LDC for tunnel disease assessment. The correlation between deformation amount, bending deformation, shearing deformation and tunnel defect in 3D-LDC of tunnels is verified through field experiments. This paper is a guide to the performance assessment based on tunnel longitudinal deformation.
(3) Comparing the accuracy of 3D-LDC and 2D-LDC indexes in tunnel defect assessment, it is shown that 3D LDC contains more accurate and comprehensive data and information. The assessment of tunnel defects requires consideration of both horizontal displacement and vertical settlement. Considering only settlement when evaluating tunnel performance may lead to overestimation of the structure performance and misjudgment of the defects.

## Figures and Tables

**Table 1:** Technical parameters of Leica ScanStaton P40.

Entry	Technical Parameters
Distance accuracy	1.2 mm + 10 ppm
Angular accuracy (horizontal/vertical)	8″/8″
Point accuracy	3 mm @ 50 m 6 mm @ 100 m
Target acquisition accuracy	2 mm @ 50 m
Dual-axis compensator	Real-time on-board liquid sensors, resolution 1″, compensation range ±5′, compensation accuracy 1.5″
Scanning range and reflectivity	120 m 8% 180 m 18% 270 m 34%
Scan Rate	1,000,000 points per second
Field of view (horizontal/vertical)	360°/290°

**Table 2:** Comparison of deformation indexes of 3D-LDC.

Type	Deformation Amount		Rotation Angle		Dislocation Amount	
	Maximum/mm	Location	Maximum/°	Location	Maximum/mm	Location
3D-LDC	727	Joint 132–133	7.23	Joint 117–118	117	Joint 117–118
Vertical LDC	681	Joint 130–131	7.22	Joint 117–118	111	Joint 117–118
Horizontal LDC	367	Joint 138–139	1.02	Joint 118–119	37	Joint 117–118

## Data Availability

Not applicable.
